# Practical Observations on Diseases of Women

**Published:** 1839-10

**Authors:** 


Art. I.—*Practical Observations on Diseases of Women.* By William Jones, M.R.C.S. *Illustrated with Cases and Explanatory Plates.* —*London,* 1839. 8vo, pp. 226.

Although we think Mr. Jones ought not to have published this book, and although we believe he will himself shortly admit, if he does not do so already, that there was no sufficient reason for his doing so, still we by no means wish to condemn it entirely. There are several useful and carefully related cases in it, but there is much of a questionable character, and there is very little that has not been abundantly published before. Mr. Jones's chief object is to magnify the utility of physical examination in uterine diseases, especially with the aid of the speculum uteri, and to urge upon those who are willing to aid in so desirable an object, the expediency of founding an institution especially devoted to the treatment of the diseases of females. The first chapter treats "of physical examination in cases of disease generally," and is a very careless chapter indeed. Thus we read that "pathology, while it teaches that alterations of structure are the causes of disease, teaches also that these alterations of structure vary *ad infinitum;* and that disease, Proteus-like, assumes a thousand varying forms, according to the numerous influences and agencies to which the human economy is subjected, each alteration being recognizable by certain physical characteristics, either during the life of the individual, or upon *post-mortem* examination." We have always been disposed to regard the alterations of structure, not as the causes of disease, but as part of the disease itself, the causes consisting in something antecedent to the change of structure. A little further on we read, "Whenever, therefore, we are called to a case of disease, we have two duties to perform, first to determine what organ it be whose altered structure gives rise to the symptoms observed; secondly, to determine the nature of the alteration which the organ may have undergone." If the preceding quotation is applied to disease generally, we can conceive nothing more incorrect; if it have any more limited application, it is carelessly indefinite in its expression. Again, "the nature of disease is to be determined by its physical symptoms, which can only be recognized through the medium of the perceptive faculties, or of what may be called physical examination." This is a simplification of the nature of disease which is quite beyond our comprehension.

Mr. Jones is an ardent advocate of the use of the speculum uteri. His advocacy, in fact, sometimes makes him incautious, for he both tells us that "he has rarely met with the objections and prejudices which some practitioners assert to exist against its use," and that "too frequently has he had to encounter the many difficulties connected with the employment of the means he advocates, to be unacquainted with the prejudices of the public respecting it." The latter statement we are more disposed to credit, and to a certain extent not to discourage the feeling whence the prejudice arises. We have on previous occasions stated our opinions respecting the use of the speculum uteri, and are not inclined to alter them. An interesting chapter is given on the history and use of the speculum, with drawings of the various forms of these instruments, which have been and still are employed, and the objections which have been urged against the employment of the instrument are considered. We are quite disposed to agree with Mr. Jones, "that the speculum is a very valuable instrument; that, by its use, information may be attained that cannot be acquired by any other means; and that, consequently, remedies may be suggested for the relief of uterine diseases, materially differing from those which would have appeared most beneficial in the absence of its employment; and hence we must admit that the practitioner who, in the present advanced state of pathological science, neglects its employment in *the cases indicating its necessity*, incurs an awful responsibility, if he be not highly culpable." We have taken the liberty of marking in italics the qualifying words in the above quotation, as we might perhaps differ from Mr. Jones in the choice of cases in which the necessity was indicated, and certainly there are cases related in his work which we feel convinced he would have treated quite as successfully without, as he did with the employment of the speculum. The following is a description of an apparatus contrived, and made use of by the author (and of which a drawing is given) for placing a patient in the most favorable position for the introduction of the instrument.

"It consists of a frame supported on four legs, to which a cushioned top is attached by hinges at one extremity; by means of these hinges and a rack placed beneath it, the top of the table may be made into an inclined plane, so as to give to the pelvis the requisite elevation; by means of a cushion for the head, those inconveniences may be obviated which might result from retaining the head in a dependant position. To prevent the legs of the patient getting in the way of the operator, two iron rods with semi-circular cushions are attached to the table, over which the legs are placed; these rods are moveable and are capable of giving different lengths, to correspond with the varied extent of the femora of different females." (p. 68.)

The book terminates by a collection of cases of various forms of disease of the generative organs, many of which are deserving of a careful perusal. In the section on amenorrhœa, the following theory of menstruation is given:

"In the earlier periods of infancy, the development of the ova is extremely slow, but as the period of puberty arrives, one or more acquires a rapid development, enlarges, and becomes distended, and making pressure upon its investing membrane, excites irritation in it and in its neighbourhood;—the termination of one of the fallopian tubes participates in and conveys that irritation to the uterus; the uterus thus excited becomes a centre of determination, and favoured by the vascularity of its tissue becomes a seat of congestion, until the minute terminations of its vessels, no longer capable of resisting distension, allow the congested fluid, somewhat altered by its passage, to escape in the form of the catamenia, meanwhile the ovum, grasped by the fimbriated extremity of the fallopian tube and becoming distended by the excitement of which itself is the centre, bursts from its confines, passes through the tube into the uterus, and subsequently is expelled, if it be not impregnated; the excitement gradually subsides after the detachment of the ovum from the ovary, and reappears at the next catamenial period, with the same train of phenomena. In support of this theory of menstruation, we have daily opportunities of remarking, at post-mortem examinations, not only that the ovaries of unmarried women bear the traces or cicatrices of detached ova, but in many instances also of proving that the number of those cicatrices corresponds with the number of catamenial effusions the individual had experienced. It has occasionally happened also that at the post-mortem examination of a woman who has died during the existence of the catamenial effusion, an ovum has been found partly within the expanded extremity of the fallopian tube, and partly detached from the ovary, whilst the ovary itself has exhibited signs of congestion, and that portion of its membrane which surrounded the ovum has borne traces of recent rupture. Some fourteen months since, this condition of the uterine appendages was witnessed by myself and Mr. Lovegrove, in the left ovary of a young woman (who died of congestion of the brain on the first day of the catamenial effusion)—in whom the hymen was perfect, and whose ovaries exhibited six or seven cicatrices; the same circumstances have been witnessed by my late colleague, Dr. Richmond, of Fenchurch street, as well as by many other individuals." (pp. 157-9.)

We have quoted the above for those who have the opportunities of so doing, to examine its correctness or the contrary. We have never seen the above-mentioned cicatrices,—possibly because we have never looked for them. This theory of Mr. Jones is an extension of that of Dr. Power, as quoted by Dr. Churchill, "that a woman menstruates because she does not conceive; that certain changes take place in the ovarian vesicles preparatory to the transmission of the ovum, and that parallel changes may take place in the uterus which may issue in the formation of the decidua;" but that, "if the stimulus of impregnation be denied, this increased action is not carried to a sufficient height to produce properly that effect; nevertheless, it is sufficient to give rise to the effusion of a fluid, *which fluid is the menstrual fluid.*"

